# Hydroxypropylsulfonation/Caproylation of Cornstarch to Enhance Its Adhesion to PLA Fibers for PLA Sizing

**DOI:** 10.3390/polym11071197

**Published:** 2019-07-17

**Authors:** Wei Li, Jie Wu, Xingui Cheng, Lanjuan Wu, Zhi Liu, Qingqing Ni, Yuhao Lu

**Affiliations:** 1 College of Textiles and Garments, Anhui Polytechnic University, Wuhu 241000, China; 2 Department of Mechanical Engineering and Robotics, Shinshu University, 3-15-1 Tokida, Ueda, Nagano 386-8576, Japan; 3 Hefei Safood Starch Co. Ltd., Hefei 230000, China

**Keywords:** hydroxypropylsulfonation/caproylation, cornstarch, past stability, adhesion, PLA fibers, film properties

## Abstract

The impact of hydroxypropylsulfonation/caproylation on the adhesion of cornstarch to polylactic acid (PLA) fibers was investigated for ameliorating the applications such as PLA sizing. The hydroxypropylsulfonated and caproylated cornstarch (HCS) samples with different degrees of substitution (DS) were synthesized by a hydroxypropylsulfonation of acid-converted cornstarch (ACS) with 3-chloro-2-hydroxy-1-propanesulfonic acid sodium salt (CHPS-Na) and subsequently a caproylation with caproic anhydride (CA). The HCS granules were characterized by Fourier transform infrared spectroscopic and scanning electron microscopy. The adhesion was evaluated by measuring the bonding forces of the PLA roving impregnated. The mechanical behaviors of the adhesive layers were estimated by determining the properties of the films. The results of adhesion measurement were also analyzed especially through the wetting and spreading of the paste on the fiber surfaces, as well as the failure type, internal stress and mechanical behaviors of the adhesive layers among fibers. Additionally, apparent viscosity and its stability of the pastes were also determined. It was found that hydroxypropylsulfonation/caproylation was not only able to obviously improve the adhesion of ACS to PLA fibers, but also capable of further improving the adhesion of hydroxypropylsulfonated starch (HS) to the fibers. With the rise in the total DS, the adhesion gradually increased. The two substituents improved the wetting and spreading, reduced the internal stress, lowered the probabilities of interfacial failure and cohesive failure, decreased the film brittleness, and increased the van der Waals force at the interfaces. Moreover, the HCS samples with a stability of above 85% could meet the demand on the stability for sizing. Considering the experimental results of the adhesion and the analysis of the results, HCS showed potential in the application of PLA sizing.

## 1. Introduction

Starch is described as one of the most abundantly occurring natural polymers [1]. It consists of both polymers known as amylose and amylopectin, which possesses a large number of α-d-glucose units connected through α-1, 4 linkages and α-1, 6 linkages [2]. It is also especially attractive due to its biodegradability [3], low cost [4,5,6], environment-friendliness, and functional properties so that starch has been widely used in the textile industry as a sizing agent and in papermaking as a surface-sizing agent [7,8]. However, the use of native starches in applications is limited because of the low resistance to shear and high temperatures and a large tendency towards retrogradation [2]. This is because the low resistance can lead to fluctuation in the viscosity of cooked starch paste [9,10], resulting in an unstable warp sizing or surface sizing, thereby affecting the quality. Starch has a tendency to retrograde and has a bad water-dispersibility, which negatively influences the wetting and spread of starch paste on the fiber surfaces, and causes a poor adhesion of starch to fibers, but also makes starch film brittle and cannot give a large deformation.

Sufficient adhesion to fibers has been perceived to be a valuable behavior for starch used for sizing in textiles and papermaking [11]. In these applications, sufficient adhesion is extremely important because adhesion, besides enhancing yarn strength, is capable of lessening the hairs of warp yarns by gluing them back onto the body of yarns during warp sizing [12]. For this reason, adhesion has become a considerably important index for accessing the quality of starch-based sizes. Additionally, the film formed at the warp surfaces is able to protect the warps from mechanical abrasions, thereby promoting the weaving ability [13]. The film is also able to enhance paper flexibility. Accordingly, the film toughness of starch occupies a very stringent place for the quality of fibrous products. Fortunately, chemical modification of starch, which involves the alteration of the physical and chemical characteristics of native starch, is usually achieved through derivatization such as etherification [14], esterification [15], and the grafting of starch [16,17], etc. The derivatization involves the introduction of functional substituents on the starch chains for altering physicochemical properties, such as shear resistance and retrogradation behavior [18]. The alteration of adhesion behavior and the toughness of starch film produced by the introduction of substituents through chemical modification have received an increasing amount of attention recently.

**Scheme 1:**
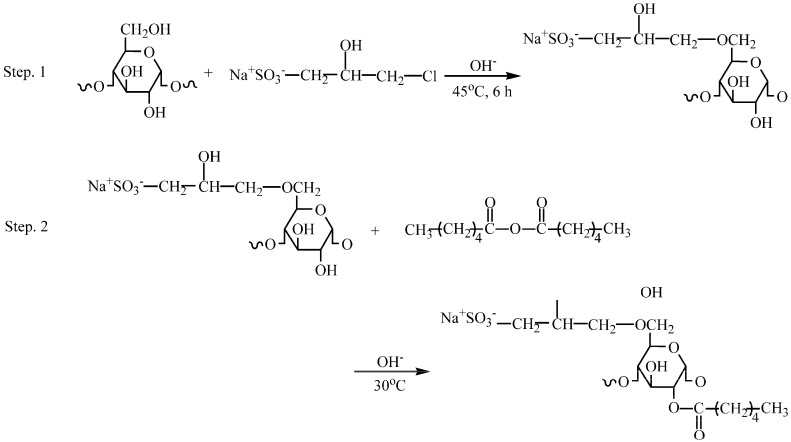
Hydroxypropylsulfonation and caproylation of corn starch for the synthesis of HCS.

Due to the similarity with the polyethylene terephthalate, eco-friendly nature, and ease of processing, polylactic acid (PLA) obtained from renewable resources has been extensively explored for fiber applications [19]. Currently, PLA warps or PLA filaments must be sized before the weaving process, due to poor cohesion, loose tows, and the easy occurrence of entanglement and bonding. Therefore, it is an urgent requirement to explore a new chemically modified starch for the use in sizing PLA warps or PLA filaments. Apparently, hydrophobic substituents that contain ester groups will favor an increase in the van der Waals forces at the interface between starch adhesive layers and PLA fibers due to the chemical similarity with the carbonyls in the PLA molecular chains, thereby being expected to improve the adhesion. However, an excessive increase in the introduction of the hydrophobic substituents can reduce the water-dispersibility and adversely affect the adhesion of starch to fibers since the paste applied for sizing warps is only a water-based adhesive. To overcome this drawback, which arises from the introduction of hydrophobic substituents, an alternative may be the introduction of hydrophobic and hydrophilic substituents into the starch molecules, simultaneously. The 3-propanesulfonic-2-hydroxy (PSH) substituents and caproates as the hydrophilic and hydrophobic substituents, respectively, can be derivatized into the starch molecules by hydroxypropylsulfonation and caproylation. The carbonyls in the caproates are chemically similar to the carbonyls in PLA molecular chains. In our previous work [20], hydroxypropylsulfonation of starch was employed to improve the adhesion of starch to cotton fibers. We found that the modification could improve the adhesion due to the improvement in the water-dispersibility of the starch after the introduction of PSH substituents. Moreover, PSH substituents and caproates with a large space volume can display strong steric restriction for interfering with the parallel arrangement of starch amyloses, partly mitigating the re-association of starch hydroxyls, and retarding the retrogradation of the paste during the formation of starch adhesive layers or starch films. Consequently, the restriction [21] can provide a plasticization for the layers or films, thereby lowering their internal stresses [22]. As a result, improved adhesion of starch to PLA fibers and a lowered brittleness of starch film may be expected, after the introduction of the PSH substituents and caproates. For these reasons, hydroxypropylsulfonation and caproylation were employed to derivatize the starch in this work. The chemical structure of hydroxypropylsulfonated and caproylated cornstarch (HCS) investigated is described in Scheme 1.

Currently, there is no study that has tested hydroxypropylsulfonation together with caproylation as the hydrophilic and hydrophobic derivatization to starch. Accordingly, an important aim of this work is to reveal if the PSH and caproate substituents chemically attached to starch chains are able to enhance the adhesion of cornstarch to PLA fibers and film properties and to determine if the HCS has a better adhesion compared with hydroxypropylsulfonated starch (HS), which has a good adhesion to cotton fibers. Another objective is to determine the optimal level of starch modification for the HCS because the functional ability of modified starch is related to the degree of substitution (DS) of the substituents introduced [23]. The obtained starch samples in this work were characterized using scanning electron microscopy (SEM) and Fourier transform infrared (FTIR) spectroscopy. Additionally, the measurements of the adhesion of cooked starch pastes to PLA fibers, light transmittance and surface tension of the pastes, tensile strength, elongation at break (%) of the films, as well as film characterization with SEM and X-ray diffraction (XRD) were also performed.

## 2. Experimental

### 2.1. Materials

Raw cornstarch (food grade) with a moisture content of 12.1 wt% and an apparent viscosity of 60 mPa·s, was obtained from Shandong Hengren Industry and Trade Co., Ltd. (Shandong, China). Prior to use, the starch was refined to get rid of the protein substance [24] and subsequently acid-converted with hydrochloric acid to decrease its excessive viscosity [25] to 23 mPa·s. The 3-chloro-2-hydroxy-1-propanesulfonic acid sodium salt (CHPS-Na) and isopropyl alcohol were offered by Jiaxing Sicheng Chemical Co. Ltd. (Jiaxing, China). Caproic anhydride (CA) used as received was supplied by Aladdin Industrial Corporation (Shanghai, China). The other analytically pure chemicals (such as sodium hydroxide, anhydrous sodium sulfate, hydrochloric acid, methanol, and ethanol) were all purchased from Sinopharm Chemical Reagent Co. Ltd. (Shanghai, China). All of the chemicals were used without further purifications before the experiments. The 100% PLA roving (520 tex), employed for adhesion measurement, was kindly provided by BBCA Group Co. Ltd. (Bengbu, China).

### 2.2. Preparation of HCS

The HCS was synthesized by reacting acid-converted cornstarch (ACS) with CHPS-Na and further caproylation with CA in an aqueous medium.

Briefly, a 40% (*w*/*w*) ACS aqueous dispersion was prepared by dispersed dried ACS (200 g) in distilled water containing Na_2_SO_4_ (20 g). The dispersion was transferred into a 1000 mL four-necked round-bottom flask, regulated at pH = 10–11 with a 3% (*w*/*w*) NaOH solution, and then heated to 45 °C in a water bath under the continuous stirring condition. Subsequently, the mixture of CHPS-Na (15 g) and NaOH (3 g) was dissolved in distilled water (30 mL) and added immediately into the dispersion. The reaction was performed at 45 °C for 6 h in the pH = 11.5–12.5 under mechanical agitation. The dispersion was neutralized to approximately pH = 7 using dilute HCl solution, vacuum-filtered, and washed three times with 75% ethanol-distilled water followed by being oven-dried at 45 °C, ground, and sieved with a 100-mesh sieve.

Subsequently, the HS prepared above was subjected to the caproylation with CA to introduce caproates into the starch molecules. The HS was dispersed in isopropyl alcohol to form a 40% (*w*/*w*) dispersion. The dispersion was regulated at pH = 8–9 using a 2% (*w*/*w*) NaOH aqueous solution. After the dispersion was heated to 30 °C under continuous agitation, CA was added slowly, meanwhile, the pH of the dispersion was maintained in a range of 8–9 by addition of the 2% (*w*/*w*) NaOH solution. After the addition, the reaction was kept at 30 °C for 0.5 h, and then the dispersion was neutralized using dilute HCl solution to approximately pH = 7. Finally, the product was vacuum-filtered, washed three times with 75% ethanol-distilled water followed by being oven-dried at 45 °C, ground, and sieved with a 100-mesh sieve for obtaining the HCS.

### 2.3. Characterization of HCS

#### 2.3.1. Measurement of DS

DS_p_ denoted the numbers of PSH substituents introduced onto the backbones of starch, and it was measured by the titration analysis [26] and calculated using the following equation:

(1) $${\mathrm{DS}}_{p}=\frac{162S}{3200-138S}$$

where 162 denotes the molecular weight of glucose unit; *S* (%) and 138 represent the sulfur content and molecular weight of –CH_2_CH(OH)CH_2_SO_3_H minus that of the hydrogen atom, respectively.

DS_c_ indicated the numbers of caproates introduced into the starch molecules. Back titration with HCl preceded by alkali saponification [27] was employed to determine the DS_c_. The caproate content (*C*, %) and its DS_c_ value were calculated with the following equations (2) and (3), respectively:

(2) $$C\left(\%\right)=\frac{(V_{1}-V_{2})\times N\times 0.099}{W}\times 100\%$$

(3) $${\mathrm{DS}}_{c}=\frac{162C}{9900-98C}$$

where *V*_1_ and *V*_2_ denote the volumes of HCl consumed in blank and sample titration; *N* is the concentration of HCl solution; 0.099 corresponds to the grams in 1 mL of 1.000 mol/L HCl solution; 9900 is the molecular weight of caproate × 100; and 98 is the molecular mass of caproate introduced minus that of hydrogen atom.

#### 2.3.2. Fourier Transform Infrared (FTIR) Spectroscopy Analysis

The infrared spectra of ACS and HCS samples were carried out on an IRPrestige-21 FTIR Spectrometer (Shimadzu Co. Ltd., Kyoto, Japan) to demonstrate the successful introduction of new functional substituents in the starch molecules after hydroxypropylsulfonation/caproylation. After the powdered ACS and HCS samples were ground with potassium bromide (KBr), respectively, and subsequently pressed into pellets, the spectra were recorded by scanning the pellets in the range from 500 to 4000 cm^−1^ with the resolution of 4 cm^−1^.

#### 2.3.3. Scanning Electron Microscopy (SEM) Analysis

The surface morphology of the granular ACS and HCS samples, as well as the cross-section of ACS, HS and HCS film samples, were analyzed by an S-4800 scanning electron microscope (Hitachi Limited, Tokyo, Japan). Prior to the analysis, all of the samples were sputter coated with a thin layer of gold to avoid charge accumulation.

#### 2.3.4. Apparent Viscosity and Viscosity Stability

The determination on the apparent viscosity of gelatinized starch paste (6% (*w*/*w*)) was performed on an NDJ-79 Rotary Viscometer (Tongji Electrical Machinery Plant, Shanghai, China) at 95 °C with a shear rate of 2028 s^−1^ (10 mPa·s ≤ viscosity values < 100 mPa·s) [28]. The data reported were the averages of two individual determination for each case. Viscosity stability was determined and calculated in accordance with the work [29].

#### 2.3.5. Surface Tension 

The surface tension of cooked starch paste was determined with a DCAT 21 Automatic Tensiometer (Dataphysics Co., Ltd., German). A 1 wt% starch aqueous paste was prepared by heating the 1% starch aqueous dispersion to 95 °C and kept at this temperature for 1 h. After cooling down, the tension was determined at room temperature.

### 2.4. Adhesion of Starch Samples to PLA Fibers

A legal standard method (FZ/T 15001-2008) was adopted to conduct the adhesion measurement. In the measurement, the bonding force of starch samples to PLA fibers was employed to indicate the adhesion. Firstly, a 1% dispersion was prepared by dispersing dried starch samples (7 g) in distilled water. The dispersion was thoroughly stirred and heated to 95 °C for 1 h to form a 1% sample aqueous paste. Afterward, the aqueous paste was used to impregnate roving that had been carefully wound onto a frame for 5 min. Then the roving impregnated was air-dried at room temperature. The dried roving was kept at 65% relative humidity (RH) and 20 °C for at least 24 h, and their bonding forces were measured using a YG026D Electronic Strength Tester (Ningbo Textile Instrument Factory, Zhejiang, China) with an initial chuck-distance of 100 mm and a drawing speed of 50 mm/min at the same ambient condition according to the literature [30]. In each case, the data presented was the average of 20 successful tests with which the abnormal values had been rejected.

### 2.5. Preparation and Property Measurement of the Films

The preparation was carried out according to our previous work [14]. The films prepared were cut into strips of 200 mm × 10 mm, and kept under above atmosphere for 24 h before the measurement.

The tensile properties were estimated via determining the tensile strength and elongation at break of the films. Before being determined, the 200 mm × 10 mm strips were maintained at 20 °C and 65% RH for 24h. The determination was carried out on the YG026D Electronic Strength Tester in an initial chuck-distance of 100 mm and a stretching speed of 50 mm/min according to the ASTM D 882-02 method. The results reported were the averages of twenty samples.

The diffraction behavior of ACS and HCS films was analyzed by XRD-6000 X-ray Diffractometer (Shimadzu Co., Japan) with a wavelength of 0.154 nm CuKa radiation at generator settings of 40 kV and 30 mA. The scanning region of diffraction was registered with a scanning rate of 4°/min and an angular step of 0.02° at Bragg angle (2*θ*) of 10° to 45°.

## 3. Results and Discussion

### 3.1. Characterization Analysis

**Figure 1:**
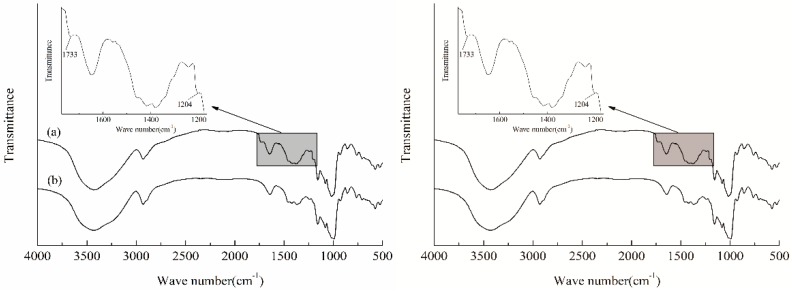
FTIR spectra of HCS (a), and ACS (b).

The introduction of PSH substituents and caproates onto starch chains was demonstrated by FTIR spectra of HCS (curve a) and ACS (curve b), as depicted in Figure 1. Besides the characteristic absorption peaks presented in the spectrum of ACS, there showed two new absorption peaks in the spectrum of HCS. The new peak appeared at the wave number of 1733 cm^−1^ was attributed to the characteristic absorption band of C=O [31] in the caproates, confirming the successful introduction of caproates in the starch molecules. The other one appeared at the wave number of 1204 cm^−1^ corresponded to the symmetric stretching vibration of sulfonic ions [32], which verified the existence of the PSH substituents on the starch chains.

**Figure 2:**
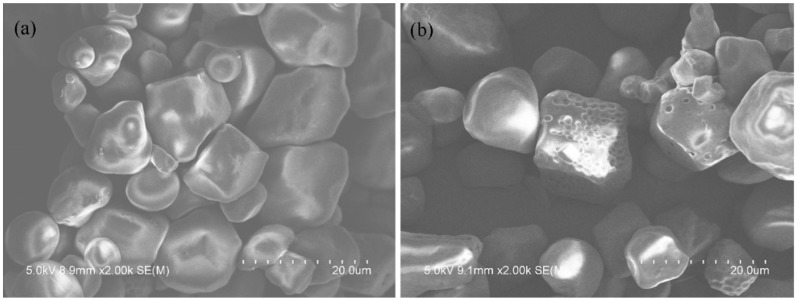
SEM images of ACS (**a**) and HCS (**b**).

It has been concluded that chemical modification is able to involve physical and chemical phenomena on the granule surface of starch [33]. SEM has been known as an important choice for clearly observing the granule morphology of modified starch and ascertaining the most substituted regions in starch granules [14]. Surface morphology of ACS and HCS granules is illustrated in Figure 2a,b, respectively. As observed in Figure 2a, it presents smooth surfaces, without obvious damage, and polygonal shapes. In contrast, there is some visible damage to the surfaces of HCS granules seen from Figure 2b. When the reaction temperature lowers the gelatinization temperature of starch, hydroxypropylsulfonation and caproylation of the granular ACS will mainly occur on the hydroxyls of granule surface under the alkaline condition. In addition, the alkaline condition is likely to cause the damage in the appearance of starch granules. As the reaction continues and damage occurs, a portion of reaction will occur in the interior of granules with the penetration of the reagents.

The total DS (contains DS_p_ and DS_c_) values of granular HCS samples are measured, as shown in Table 1. As expected, when the DS_p_ was a fixed 0.029 prepared by reacting ACS with 15 g of CHPS-Na, with the increases in the amount of CA to starch, the DS_c_, i.e., level of starch caproylation, gradually increased. Therefore, it could be imagined that HCS samples with gradually increased total DS (the sum of DS_p_ and DS_c_) values from 0 to 0.087 could be obtained as the amount of the CA increased.

### 3.2. Impact of Hydroxypropylsulfonation/Caproylation

#### 3.2.1. Impact on Apparent Viscosity and Its Stability

**Figure 3:**
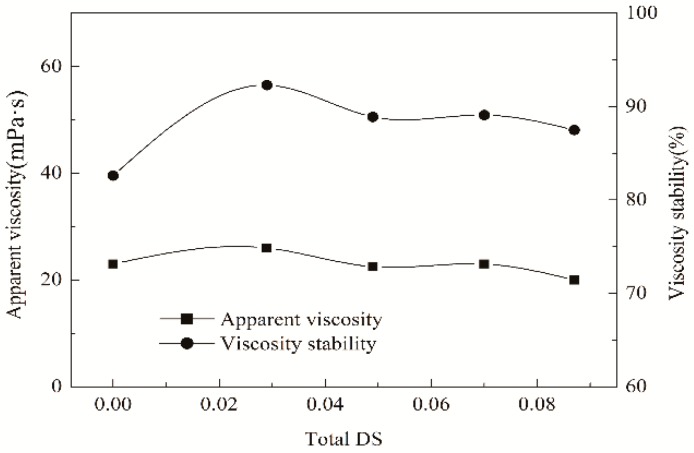
Apparent viscosity and its stability of gelatinized starch paste.

The impact of the PSH substituents and caproates introduced onto ACS chains upon apparent viscosity and its stability of cooked ACS paste is showed in Figure 3. As found, after the introduction of the two substituents, the paste viscosity did not show an obvious difference with that of the ACS (DS = 0) and HS (DS = 0.029). In addition, with the increases in DS values, i.e., the amounts of the PSH substituents and caproates introduced, the variation of the viscosity was unobvious. Moreover, the HCS samples were superior to their counterpart ACS in the stability of paste viscosity but were inferior to HS sample in stability. As the total DS raised, the stability exhibited a decreased tendency. It suggested that the introduction of caproates led to a negative impact on the stability. Fortunately, in the total DS range of 0.049–0.087, the stability was above 85%. In general, the viscosity stability of the paste required is ≥ 85% during its use in sizing [34]. Consequently, the introduction of the substituents can meet with the demand on the stability for warp sizing.

As is well known, starch granules cooked in its aqueous dispersion at high temperature will swell by adsorbing the water molecules, and the crystalline structure of the granules will be destroyed to form an amorphous structure; subsequently, the dispersion converts into a gelatinized starch paste [35]. The paste produced can be described as a biphasic system of simultaneously containing disperse and continuous phases [36]. The disperse phase is described as swollen remnants of the granules (mainly consists of the amylopectin) and a continuous one, known as a solution of the soluble starch coins (mainly composed of amyloses) [37]. Apparently, the increase in the apparent viscosity after the introduction of the PSH substituents is not only attributed to the hydrophilicity and ionization of the substituents but also attributed to the greater expansion of soluble starch coils and swelling of the remnants. The hydrophilicity can enhance the intermolecular interaction between starch and water, and the ionization can induce the repulsion between starch chains, thereby causing greater expansion and further swelling. The greater expansion and further swelling raise the kinetic volumes of the swollen remnants and coils, producing an increased resistance to paste flow [21]. Accordingly, the viscosity of HS is found to increase compared with that of ACS. The slight decrease in the viscosity after the introduction of the caproates onto starch chains is mainly due to hydrophobicity and nonionization. The nonionization does not produce the repulsion, and the hydrophobicity can reduce the intermolecular interaction between starch and water, thereby being probable to restrain the swelling, and producing a slight decrease in the viscosity of HCS compared with HS.

The starch paste must be stable in viscosity during warp sizing because high stability favors the stability of size pick-up [14], thereby enhancing weaving efficiency. It is well known that d-glucosidic bonds are sensitive to the heat and shear where they will break and degrade the starch chains in the cooked aqueous paste, eventually producing the fluctuation in the viscosity [38]. The fluctuation causes a lowered stability. Therefore, the ACS paste has a stability of 82.6%. Nevertheless, the introduction of the hydrophilic PSH substituents significantly ameliorates the stability. It may be attributed that the hydrophilicity promotes interaction and affinity between starch molecules and water, and lowers the degradation, and the hindrance obstructs the hydrogen bonding between starch hydroxyls and alleviates the aging of the paste. The decrease in the stability after the introduction of the hydrophobic caproates is possibly attributed that the hydrophobic caproates reduce the interaction and affinity between starch molecules and water, and the degradation extent is probable to enhance. However, it can be found from the Figure 3, all of the HCS samples have the stability of above 85% in the total DS range of 0.049–0.087, thereby confirming the stability of size pick-up during the sizing process.

#### 3.2.2. Impact on Adhesion

**Figure 4:**
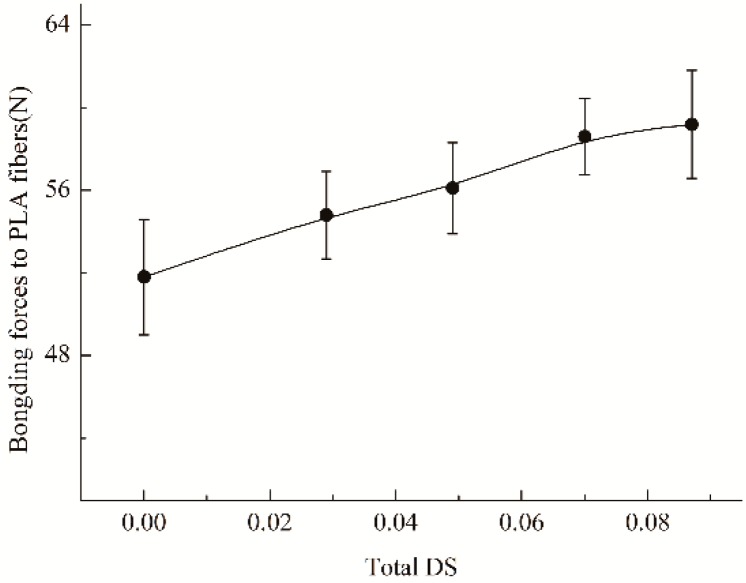
Impact of hydroxypropylsulfonation/caproylation on adhesion of starch to PLA fibers.

As is well known, adhesion of sizing or coating agents to fibers displays the effect of increasing the strength of fibrous goods by bonding the fibers together. Hence, the impact of hydroxypropylsulfonation/caproylation on the adhesion should be estimated, and the results as a function of DS value of the substituents, the average number of the substituents introduced per anhydroglucose unit of starch, are represented in Figure 4. It could be found that HCS was superior to HS (total DS = 0.029) and ACS (total DS = 0) in the bonding forces to PLA roving. This observation implies that hydroxypropylsulfonation/caproylation is not only able to obviously improve the adhesion of ACS to PLA roving but also capable of further ameliorating the adhesion of HS to PLA roving. The forces of HCS were correlated with the total DS. As the DS increased, the forces gradually increased, and when the DS was above 0.070, the force just had a slight increase.

According to the failure position, the failure of an adhesive joint usually includes cohesive failure and interfacial one [39]. The former denotes a failure that occurs wholly within the matrix of an adhesive layer formed by adhesives such as starch. The latter indicates a failure that occurs exactly at the interfaces between the layers and fibers. The fracture of an adhesive joint commonly occurs at its weakest area, and thus the bonding forces of the joint can be estimated by mechanical properties of the adhesive layers and interfacial attraction. Apparently, a strong bonding can be achieved only when there are no occurrences of both cohesive failure and interfacial failure at low applied loads. To investigate the impact of chemical modification on the adhesion, the prime consideration will be the interfacial interactions at the interfaces, subsequently considering the mechanical properties of the adhesive layers formed by the paste covered at the fiber surfaces.

Owing to the reassociation of hydroxyls between linear amyloses, the amyloses in the low-temperature aqueous paste are inclined to regularly arrange themselves and form the aggregates [40]. Additionally, amylose molecules can also co-crystallize with the linear branches of amylopectin [41] through the association of hydroxyls [42]. The aggregates and co-crystallization can result in the retrogradation of starch paste and make the paste microheterogeneous, inevitably inducing an incomplete wetting and outspreading of the paste onto the fiber surface [30]. It has been verified that incomplete wetting and outspreading are deleterious to adhesion [43] because the interface failure and high internal stresses are easy to occur around unwetted or outspread areas. Moreover, starch paste that exists around the surface of fibers, will shrink in the volume with the loss of water during drying, subsequently converting into adhesive layers between the fibers in the roving. Due to the brittleness characteristic of starch, the shrinkage will bring about high internal stresses at the interfaces and within the bulk phase of the layers [30]. Local interfacial failure will occur once the local stress exceeds the local strength. It has been confirmed that high internal stresses can impose intensive damage on the adhesion [43].

**Figure 5:**
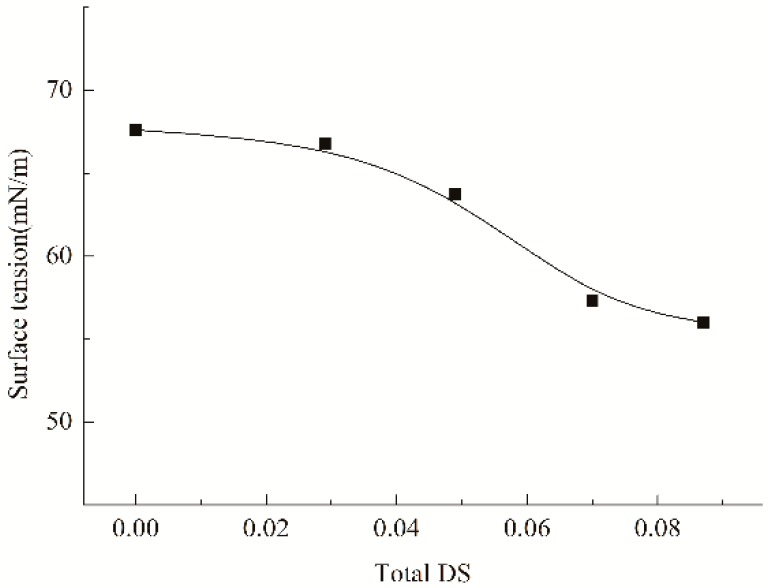
Impact of the caproates and PSH substituents on the surface tension of cooked starch paste.

**Figure 6:**
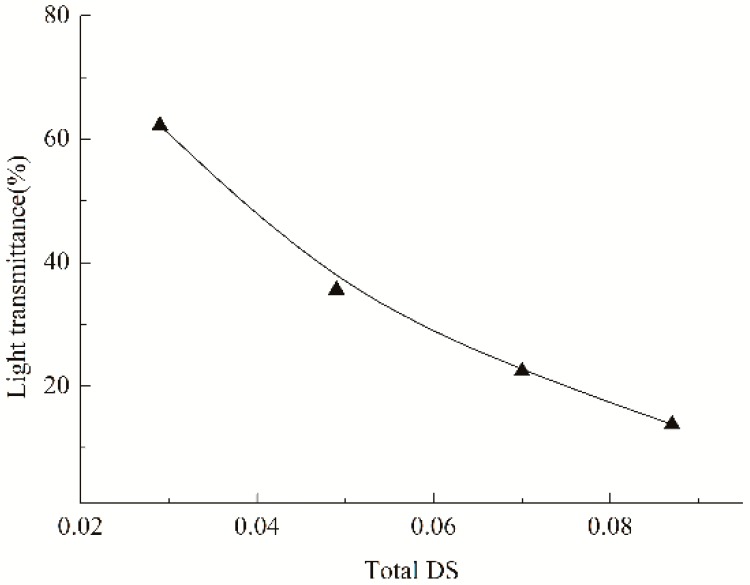
Light transmittances of cooked HCS pastes.

Obviously, the substituents introduced can interfere with the re-association of the hydroxyls and alleviate the parallel arrangement of the amyloses through their steric hindrance, thereby favoring to alleviate the retrogradation [14]. As a result, an amelioration in the wetting and spreading may be expected. Furthermore, as broadly accepted, wetting and spreading abilities of an adhesive liquid onto a given solid surface is correlated with its surface tension [30]. A reduced tension favors the wetting and spreading, and often induces an improved adhesion [44]. For the reason, the impact of caproates and PSH substituents on the surface tension of cooked starch paste was measured, as depicted by indicating the tension versus total DS values in Figure 5. It was observed that the two substituents gave much impact on the tension, and the tension decreased after the introduction of the two substituents. The tension depended on the total DS, and it gradually reduced in the DS range of 0.049–0.087. In addition, the HCS pastes had lower tension than ACS and HS had. This means that caproates and PSH substituents are both necessary to provide the derivatives with stronger surface activity. A decreased tension also favors to ameliorate the wetting and spreading of cooked starch pastes onto the fiber surfaces. The amelioration will lower the probability of interfacial failure mentioned above, providing an improvement in the adhesion. Apparently, the hindrance of caproates and PSH substituents is larger than that of PSH substituents, subsequently exhibiting a stronger obstruction to the re-association and a greater alleviation in the arrangement. Additionally, the HCS paste has a lower surface tension compared to the HS paste. Therefore, the HCS is superior to HS in the adhesion. Besides, hydrophobic caproates that contain ester groups will favor to increase the van der Waals force at the interfaces between starch adhesive layers and PLA fibers, due to the chemical similarity with the carbonyls in PLA molecular chains, thereby also favoring the improvement the adhesion. These factors reinforce the interfacial actions and provide a positive effect on the adhesion. However, the introduction of the hydrophobic caproates will reduce the water-dispersibility of starch, which can be evaluated by the measurement of the light transmittance of the paste, as depicted in Figure 6. As seen, with the increase from 0.029 to 0.087 in the total DS (from 0 to 0.058 in the DS_c_ of the caproates introduced), light transmittances of the starch pastes gradually decreased. Commonly, the lower the transmittance, the worse the water-dispersibility [45]. Therefore, it can be concluded that water-dispersibility of the HCS paste continuously reduces as the amount of the caproates increases. It has been demonstrated that the worse water-dispersibility leads to incomplete wetting and outspreading [15], thereby resulting in the occurrence of the interfacial failure and generating an adverse effect on the adhesion. Therefore, the combination of the positive and negative factors may be expected to reduce the probability of interfacial failure, favoring the adhesion. Based on the reduced probability, the adhesion of the HCS to fibers will increase if the cohesive force of the HCS adhesive layer is strong enough.

**Figure 7:**
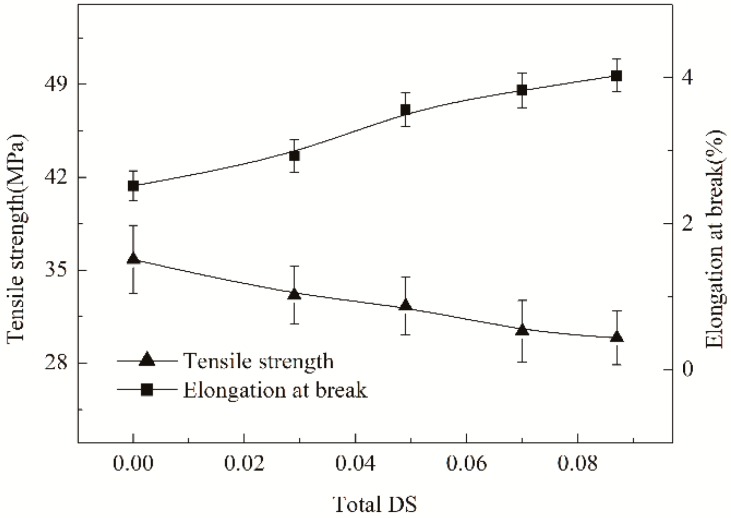
Impact of hydroxypropylsulfonation/caproylation on tensile strength and elongation at break of ACS films.

**Figure 8:**
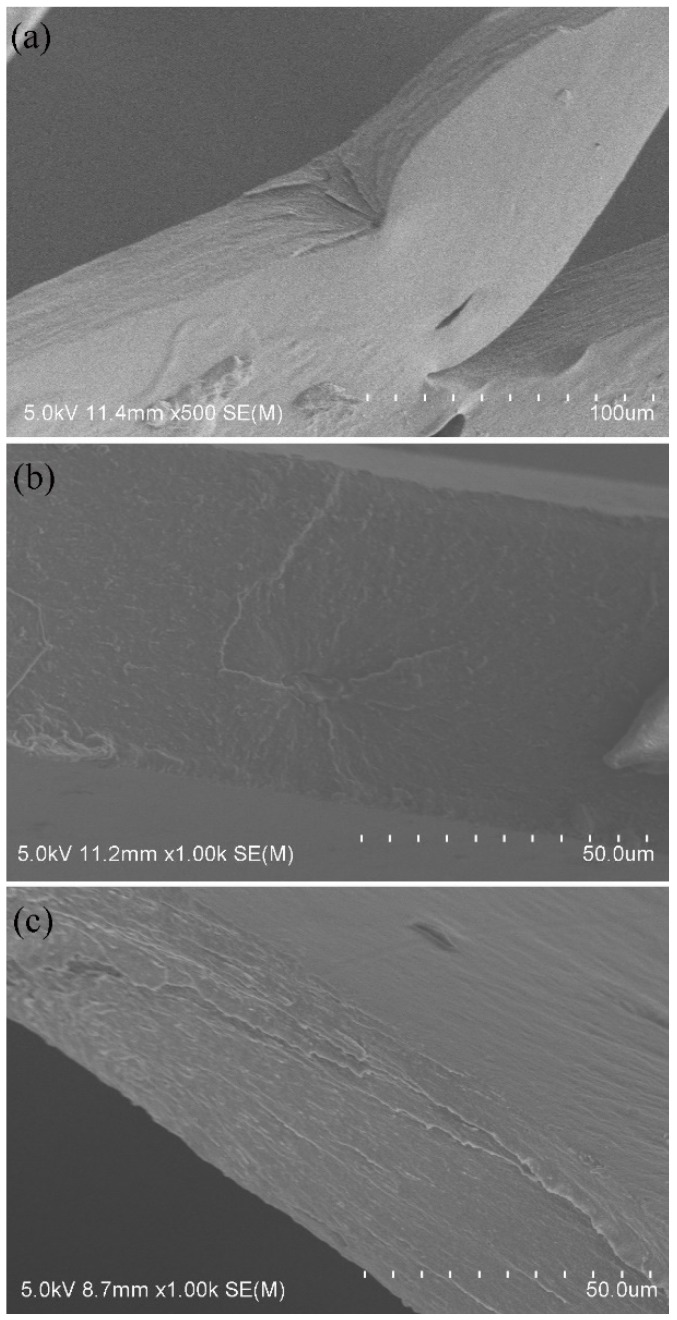
SEM images of the cross sections of the ACS (**a**), HS (**b**) and HCS (**c**) films.

In general, the cohesive failure of an adhesive joint is also correlated with the mechanical properties of the adhesive layers existed among fibers [25]. Accordingly, the properties should be investigated for the estimation of the cohesive failure. Nevertheless, it should be clearly noted that it has considerable difficulty to strip the adhesive layers among fibers for determining the real mechanical properties of the layers. Fortunately, the HCS films cast from cooked HCS pastes are capable of being adopted to estimate the properties such as tensile strength, elongation at break, degree of crystallinity and flexibility. The tensile strength and elongation at break are determined and shown in Figure 7. Compared with ACS film, the strength of the HCS films decreased, but the elongation increased. The strength and elongation depended on the total DS. In addition, with the rise in the total DS, the elongation gradually promoted whereas the strength continuously lowered. This observation suggests that the hydroxypropylsulfonation/caproylation can reduce the brittleness of starch films or adhesive layers, exerting a toughening effect on the films or adhesive layers. The toughening effect provided by the modification can be verified by observing the SEM images of the cross-section of ACS, HS and HCS film samples, as represented in Figure 8a–c, respectively. It could be seen from Figure 8a that ACS film exhibited a strong brittleness while HS and HCS films displayed a lower brittleness seen from Figure 8b and c, respectively. This observation means that the hydroxypropylsulfonation/caproylation produces a toughening effect on the film. Undoubtedly, the HCS film (described as HCS adhesive layers that formed among PLA fibers) with a lower brittleness is preferable to the brittle ACS film for the improvement of the adhesion, so as to promote the applications of HCS in warp sizing and surface coating.

**Figure 9:**
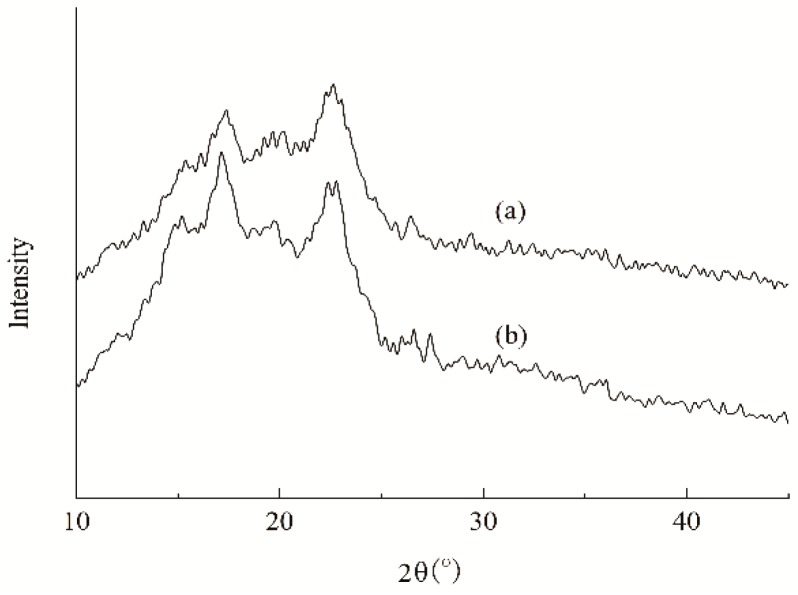
X-ray diffractograms of the HCS (a) and ACS (b) films.

During the formation of the film or the adhesive layer, the amylose chains in the aqueous paste regularly arrange with each other by hydrogen bonding between the hydroxyls and form a three-dimensional network structure [46]. It is the most important reason that starch film exhibits a strong brittleness and cannot produce a large deformation. The PSH substituents and caproates act like the wedges between starch chains through their hindrance. Consequently, the substituents favor disturbing the arrangement in an orderly way of the amyloses during film or adhesive layer formation. As a result, a lowered degree of crystallinity ascertained by X-ray diffraction of the starch film can be expected and is depicted in Figure 9. Compared with X-ray diffractogram of ACS film, the HCS film was smaller than ACS film in the areas of crystalline regions. It implies that the HCS film has a lower crystallinity, which is mainly attributed to the hindrance of the substituents introduced diminishes the inter-chain hydrogen bonding and exhibits the disturbance to the regular arrangement of starch chains, subsequently lowering the formation of the crystalline structure. Furthermore, the hindrance of the two substituents can also provide an internal plasticization for the layers. Consequently, a higher elongation and a lower strength for the HCS film are displayed. Undoubtedly, an adhesive layer with a lower brittleness will avoid the occurrence of strong internal stresses in the layer, thereby diminishing the probability of cohesive failure within the matrix of the adhesive layer, and favoring the adhesion.

## 4. Conclusions

Based on the previous results and discussions, it could be concluded that introducing caproates and PSH substituents into starch molecules via hydroxypropylsulfonation and caproylation of ACS with CHPS-Na and CA was an effective manner to improve the adhesion of starch to PLA fibers for ameliorating its end-use ability in the applications such as PLA warp sizing and papermaking. The bonding forces of HCS (total DS = 0.049–0.087) to PLA fibers were superior to those of ACS (DS = 0) and HS (DS = 0.029), indicating hydroxypropylsulfonation/caproylation was not only able to improve the adhesion of starch to PLA fibers, but also able to further improve the adhesion compared with the HS. The adhesion of HCS to PLA fibers was correlated with the total DS, and the forces gradually increased as the total DS raised. In addition, all of the HCS samples had the stability of above 85%, indicating that the introduction of the substituents could meet with the requirement in the stability for warp sizing. The elongation at break of the HCS film was higher than ACS film, and tensile strength and degree of crystallinity of the film were inferior to those of ACS. These suggested that caproates and PSH substituents derivatized were capable of reducing the brittleness of the film, and providing a toughening effect on the film adopted to evaluate the mechanical properties of HCS adhesion layers. The toughening effect provided by the two substituents indicated that they are capable of reducing the internal stresses within the matrix of the adhesive layers, thereby diminishing the probability of cohesive failure within the matrix of the adhesive layer, and favoring the adhesion. Moreover, the substituents not only favor alleviating the retrogradation through interfering with the re-association of the hydroxyls and alleviating the parallel arrangement of the amyloses but also favor reducing the surface tension of cooked starch paste; thereby, favoring the amelioration in the wetting and spreading and subsequently lowering the probability of interfacial failure. Hydrophobic caproates that contain ester groups also favor increasing the van der Waals force at the interfaces between starch adhesive layers and PLA fibers. For these reasons, the modification improved the adhesion. Considering the results of HCS in the paste stability, adhesion, film properties, as well as preparation cost, thus HCS with a total DS of 0.070 showed potential in the applications of PLA sizing.

## Figures and Tables

**Table 1:** DS of starch modification, i.e., the hydroxypropylsulfonation (DS_p_) and caproylation levels (DS_c_).

Starch Samples	Weight of CHPS-Na(g)	DS_p_	Weight of CA(g)	DS_c_	Total DS
ACS	0	-	0	-	-
HS	15	0.029	0	-	0.029
HCS	15	0.029	7.5	0.020	0.049
	15	0.029	15.0	0.041	0.070
	15	0.029	22.5	0.058	0.087

## Abbreviations

polylactic acid	PLA
hydroxypropylsulfonated and caproylated corn starch	HCS
degree of substitution	DS
acid-converted corn starch	ACS
3-chloro-2-hydroxy-1-propanesulfonic acid sodium salt	CHPS-Na
caproic anhydride	CA
Scanning electron microscopy	SEM
hydroxypropylsulfonated starch	HS
3-propanesulfonic-2-hydroxy	PSH
Fourier transform infra-red	FTIR
X-ray diffraction	XRD
relative humidity	RH
